# Practice and attitudes of infection control staff towards diagnostic stewardship measures

**DOI:** 10.3205/dgkh000601

**Published:** 2025-11-28

**Authors:** Sebastian Schulz-Stübner, Teresa Tamayo

**Affiliations:** 1 German Consulting Center for Infection Prevention and Control, Freiburg i.Brsg., Germany; 2 Clinic for Anesthesiology and Critical Care, University Medical Center Freiburg, University Freiburg, Freiburg i. Brsg., Germany; 3 University of Education, Freiburg i. Brsg., Germany

**Keywords:** diagnostic stewardship, antibiotic stewardship, infection control team

## Abstract

**Introduction:**

In recent years, diagnostic stewardship has gained importance worldwide as part of antibiotic stewardship and infection control programs. However, the specific involvement of infection control (IC) teams in this area has not been studied.

**Method:**

A volunteer survey of participants at the 2024 Freiburg Conference on Infection Prevention and Therapy was conducted to assess attitudes and practices regarding diagnostic stewardship.

**Results:**

The majority of the 182 participants worked in German hospitals with established IC-committees (91.2%), antibiotic stewardship teams (43.4%), and laboratory commissions (24.7%).

For sepsis diagnosis, at least two pairs of blood cultures are usually taken, which is in line with the guidelines; 14.3% use the “six-pack” rule (three pairs), and 28.6% take all cultures from one puncture site. For many clinical tests –except of stool tests and C-reactive protein – less than 50% rated their use as “appropriate”, indicating a need for improvement. Interleukin 6 and beta-D-glucan are rarely used.

Strategies such as reflex tests and cascade reporting are only used occasionally and are viewed with scepticism in some cases. Screening for methicillin resistant *Staphylococcus* *aureus* and Vancomycin resistant enterococci was rated as “appropriate” by over 60%, while screening for multidrug-resistant Gram-negative bacteria was rated as such by 50%. In the area of IC, 32.4% reported inadequate sampling of surfaces and 33.2% of staff hands.

**Discussion:**

Subjective assessments and a heterogeneous participant structure limit the survey, and subgroup analyses are not possible due to the small number of cases. However, the results show chances for education and integration of IC teams in diagnostic stewardship programs.

## Zusammenfassung

**Einleitung:**

In den letzten Jahren hat das so genannte Diagnostic Stewardship im Rahmen von Antibiotic Stewardship- und Infektionskontrollprogrammen weltweit an Bedeutung gewonnen. Die spezifische Einbindung der Hygieneteams in diesen Bereich ist jedoch wenig untersucht.

**Methode:**

Im Rahmen einer Umfrage unter Teilnehmenden der Freiburger Infektiologie- und Hygienekongresses 2024 wurden Einstellungen und Praktiken zum Thema Diagnostic Stewardship erfasst.

**Ergebnisse:**

Die Mehrheit der 182 Teilnehmenden arbeitete in deutschen Krankenhäusern mit etablierten Hygienekommissionen (91,21%), Antibiotic Stewardship-Teams (43,41%) und Laborkommissionen (24,73%).

Bei der Sepsisdiagnostik werden meist mindestens zwei Blutkulturpaare entnommen, was den Leitlinien entspricht; 14,29% nutzen die „Six-Pack“-Regel (drei Paare), 28,57% nehmen alle Kulturen aus einer Punktionsstelle ab. Bei vielen klinischen Tests – mit Ausnahme von Stuhluntersuchungen und CRP – bewerteten weniger als 50% die Nutzung als „angemessen“, was auf Verbesserungsbedarf hinweist. IL-6 und Beta-D-Glucan werden selten verwendet.

Strategien wie Reflex-Tests und gesteuerte Antibiogramme werden nur gelegentlich verwendet und teils skeptisch betrachtet. Das Screening auf Methicillin resistente *Staphylococcus* *aureus* und Vancomycin resistant Enterokokken wurde von über 60% als „angemessen“ bewertet, beim Screening auf multiresistente Gram-negative Erreger von 50%. Bei den hygienespezifischen Fragen bewerten 32,4% eine Probenahmen von Oberflächen und 33,2% von den Händen des Personals als „zu wenig“.

**Diskussion:**

Die Umfrage ist durch die subjektiven Einschätzungen der Teilnehmenden und eine heterogene Teilnehmerstruktur limitiert, zudem sind Subgruppenanalysen aufgrund geringer Fallzahlen nicht möglich. Die Ergebnisse zeigen Verbesserungspotential hinsichtlich der Einbindung der Hygieneteams beim Thema Diagnostic Stewardship und auch bei der Probenahme von Oberflächen und Händen.

## Introduction

In recent years, diagnostic stewardship has gained attention as part of antibiotic stewardship and infection control programs worldwide [1], [2], [3], [4], [5]. However, the involvement of infection control (IC) staff in these activities remains unclear and is not reported in the literature.

## Method

To analyse the practice and attitudes regarding diagnostic stewardship among members of IC teams, we conducted a survey among visitors during the annual Freiburg conference of infection prevention and therapy 2024. Each participant agreed to take the survey and data-sheets (Attachment 1) were collected anonymously in drop-off-boxes.

## Results

Table 1 (Tab. 1) shows the summary of the survey’s questions and the frequency of responses. Most of the 182 participants worked in German hospitals. Respondent’s institutions had an IC board, antibiotic stewardship teams, and a lab-commission specialized in diagnostic tests in 91.2%, 43.4%, and 24.7% respectively.

In most institutions a minimum of two pairs of blood cultures (BC) are taken for diagnosis of sepsis which is in accordance with current clinical guidelines. 14.3% have established the “six pack” rule (three pairs of BC) and 28.6% report collecting all blood cultures from a single puncture as recommended by emerging literature. 

For most clinical test items with exception of stool testing and CRP, less than 50% of respondents felt that the utilization is “just right”, indicating relevant potential for improvement. IL 6 and Beta-D-Glucan are rarely utilized.

Diagnostic stewardship strategies like reflex testing and cascade reporting of resistance profiles are used only occasionally and sometimes met with scepticism.

Screening for MRSA and VRE was judged as “just right” by more than 60% of respondents, screening for multiresistant Gram-negative bacteria by 50%. 

In the IC-related questions, 32.4% of respondents consider the amount of sampling to be insufficient regarding surface sampling while 33.2% believe the same for sampling from hands of staff. 

## Discussion

While the relatively high number of “unclear” answers in the specific test related questions can be explained by lack of involvement of the IC-Team members in clinical decision making, it also demonstrates the need for a more integrative approach between antibiotic stewardship and infection prevention. 

The large number of respondents considering microbiological hand sampling as “not enough” came as a surprise considering much better educational tools for hand hygiene monitoring and motivation like real time fluorescent or dye-based visualization techniques; similarly, environmental surface sampling was often regarded as insufficient, although routine environmental sampling of surfaces is not recommended [6]. Van der Schoor et al. [7] conducted a web-based survey regarding environmental sampling in which most respondents were clinical microbiologists or infection prevention and control practitioners, and 57.3% were from either the Netherlands, the United Kingdom, or Ireland. Respondents had high self-reported knowledge, which was not consistent with their response to certain questions. There was no consensus on sample sites, neither within nor between countries [7]. Obviously, the same uncertainty exists in Germany.

## Conclusion

The results indicate that German IC teams need more education and practical involvement in diagnostic stewardship activities not only in the context of antibiotic stewardship but also in their own field, given the high levels of uncertainty regarding environmental samples and hand hygiene related sampling.

## Limitations

Our study is limited by the subjective nature of the answers and the large variety of professions. Overall, numbers in surveyed healthcare professions were too small for subgroup-analyses.

## Notes

### Authors’ ORCIDs

- Schulz-Stübner S: 0000-0001-5210-9364

### Ethical approval 

At a conference, participants agreed to participate in the voluntary anonymous survey.

### Funding

This work was funded by institutional funds only.

### Competing interests

The authors declare that they have no competing interests.

## Supplementary Material

Survey items

## Figures and Tables

**Table 1:**
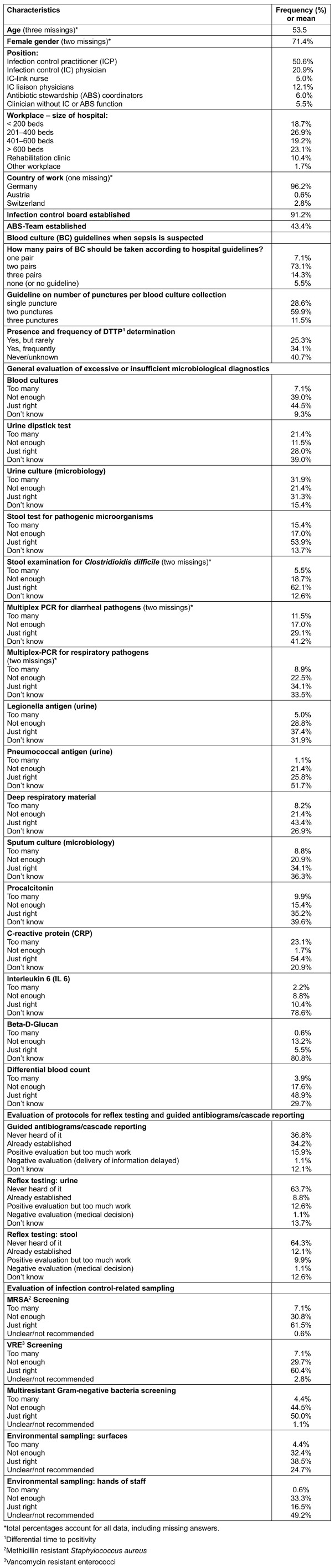
Surveyed characteristics of participants and their evaluation of infection control and diagnostic stewardship guidelines and activities (n=182*)
