# Supplementary material for: Practice and attitudes of infection control staff towards diagnostic stewardship measures
Source: GMS Hyg Infect Control. 2025 Nov 28;20:Doc72. doi: 10.3205/dgkh000601 (PMC12720261; doi:10.3205/dgkh000601)
Supplement: Survey items [file HIC-20-72-s-001.pdf]

Attachment 1: Survey items

A Experience regarding „Diagnostic Stewardship“ in your institution

1. **Where have you discussed „Diagnostic Stewardship“-related items? (please consider the last 12 months only)**

If you work in more than one facility answer for the one you spent most of your time!.

	Does not exist	rarely	sometimes	often
Infection Control Committee	☐	☐	☐	☐
Antibiotic Steering Committee	☐	☐	☐	☐
Antibiotic Stewardship-Team	☐	☐	☐	☐
Laboratory Committee	☐	☐	☐	☐
Others:				

B What is your personal experience regarding „Diagnostic Stewardship“

2. **Have you been contacted in the last 12 months from healthcare workers in your facility regarding "<u>Diagnostic Stewardship</u>"?**

If you work in more than one facility answer for the one you spent most of your time!.

☐ no ☐ yes ►who ____

► what topic____

3. Ho many blood cultures do your institutional guideline for sepsis work-up suggests?

If you work in more than one facility answer for the one you spent most of your time!

☐ 1 pair (aerob/anaerob)
☐ 2 pairs (aerob/anaerob)
☐ 3 pairs (aerob/anaerob)
☐ We don't have a guideline

Comments:

3.1. Are you using the „differential-time to positivity" to diagnose catheter-related blood stream infections?

☐ yes but only in special cases
☐ yes, often
☐ no, never
☐ Don't know
☐ Others: ____________________

3.2. What is your routine to take blood cultures?

☐ Single site sampling (e.g. 60 ml für three pairs from one puncture)
☐ Multisite sampling with each pair of cultures filled separately from a different puncture site
☐ Don't know
☐ Others: ____________________

3.3 Do you know the rejection criteria of your microbiology lab for the following materials?

Sputum		Urine		Stool	
Yes	no	Yes	No	Yes	No
☐	☐	☐	☐	☐	☐

Comments:

4. Are urine cultures taken preoperatively in non-urological surgical cases?

☐ yes ☐ no ☐ sometimes ☐ Don' know

If yes, which surgeries:

C Your opinion regarding specific „Diagnostic Stewardship“-interventions

5. What do you think about the cascade reporting of resistance profiles?

☐ Never heart of
☐ Current practice
  ☐ good acceptance ☐ bad acceptance
☐ Would like to have but to cumbersome
☐ Not acceptable, because important information will not be readily available
☐ Don' know

6. What do you think about „Reflex-Testing“ and fixed stop-criteria for urine samples?

☐ Never heart of
☐ Current practice
  ☐ good acceptance ☐ bad acceptance
☐ Would like to have but to cumbersome
☐ Not acceptable because it interferes with freedom of practice
☐ Don' know

7. What do you think about „Reflex-Testing“ and fixed stop-criteria for stool samples?

☐ Never heart of
☐ Current practice
  ☐ with good acceptance ☐ with bad acceptance
☐ Would like to have but to cumbersome
☐ Not acceptable because it interferes with freedom of practice
☐ Don' know

D Your experience regarding microbiological sampling and biomarker tests

8. How do you judge microbiological sampling practices in your institution in the last 12 months regarding the following items
If you work in more than one facility answer for the one you spent most of your time!.

Clinical indication:

Blodd culture?
☐ too much ☐ too little ☐ just right ☐ Don' know ☐ not done

Urine dipp stick test?
☐ too much ☐ too little ☐ just right ☐ Don' know ☐ not done

Urine culture?
☐ too much ☐ too little ☐ just right ☐ Don' know ☐ not done

Stool culture?
☐ too much ☐ too little ☐ just right ☐ Don' know ☐ not done

Stool for *Clostridioides difficile*-toxine?
☐ too much ☐ too little ☐ just right ☐ Don' know ☐ not done

Stool with multiplex-PCR?
☐ too much ☐ too little ☐ just right ☐ Don’ know ☐ not done

Respiratory material with multiplex PCR?
☐ too much ☐ too little ☐ just right ☐ Don’ know ☐ not done

Legionella-antigen test in urine?
☐ too much ☐ too little ☐ just right ☐ Don’ know ☐ not done

Pneumococcal-antigen test in urine?
☐ too much ☐ too little ☐ just right ☐ Don’ know ☐ not done

Tracheal aspirate or BAL for culture?
☐ too much ☐ too little ☐ just right ☐ Don’ know ☐ not done

Sputum for culture?
☐ too much ☐ too little ☐ just right ☐ Don’ know ☐ not done

PCT?
☐ too much ☐ too little ☐ just right ☐ Don’ know ☐ not done

CRP?
☐ too much ☐ too little ☐ just right ☐ Don’ know ☐ not done

IL 6?
☐ too much ☐ too little ☐ just right ☐ Don’ know ☐ not done

Beta-D-Glucan?
☐ too much ☐ too little ☐ just right ☐ Don’ know ☐ not done

Differential blood count?
☐ too much ☐ too little ☐ just right ☐ Don’ know ☐ not done

Infection prevention indications:

MRSA-Screening?
☐ too much ☐ too little ☐ just right ☐ Don’ know

VRE-Screening?
☐ too much ☐ too little ☐ just right ☐ Don’ know

Multiresistant GNB-Screening?
☐ too much ☐ too little ☐ just right ☐ Don’ know

Routine microbiological sampling of surfaces?
☐ too much ☐ too little ☐ just right ☐ Don’ know ☐ not done

Routine microbiological sampling of Health Care Workers hands?
☐ too much ☐ too little ☐ just right ☐ Don’ know ☐ not done

Attachment 1 to: *Schulz-Stübner S, Tamayo T. Practice and attitudes of infection control staff towards diagnostic stewardship measures. GMS Hyg Infect Control. 2025;20:Doc72. DOI: 10.3205/dgkh000601*

E Demographics

9. **Age** ________(years) **Gender** ☐ female ☐ male ☐ divers

10. **Your professional activity (multiple answers possible)**

☐ Infection Control Practitioner
☐ Link Nurse
☐ Hospital Epidemiologist
☐ Infection Control Liaison physician
☐ Occupational Medicine
☐ Public Health Specialist
☐ ABS-Liaison physician
☐ Clinical Physician
☐ Others____

11. **Describe the facility for which you have answered?**

Hospital:
☐ less than 200 beds ☐ 201-400 beds ☐ 401-600 beds ☐ more than 600 beds
☐ Rehabilitation-Hospital
☐ Outpatient surgical centre
☐ Others____
in
☐ Germany ☐ Austria ☐ Switzerland ☐ Others___________________________

12. **How do you work in that facility?**

☐ employed ☐ external contractor ☐ Others___________________________
